# Supplementary material for: Molecular Determinants of TMC Protein Biogenesis and Trafficking
Source: Int J Mol Sci. 2025 Jul 1;26(13):6356. doi: 10.3390/ijms26136356 (PMC12250529; doi:10.3390/ijms26136356)
Supplement: Supplementary file 1 [file ijms-26-06356-s001.zip › Supplementary Materials.pdf]

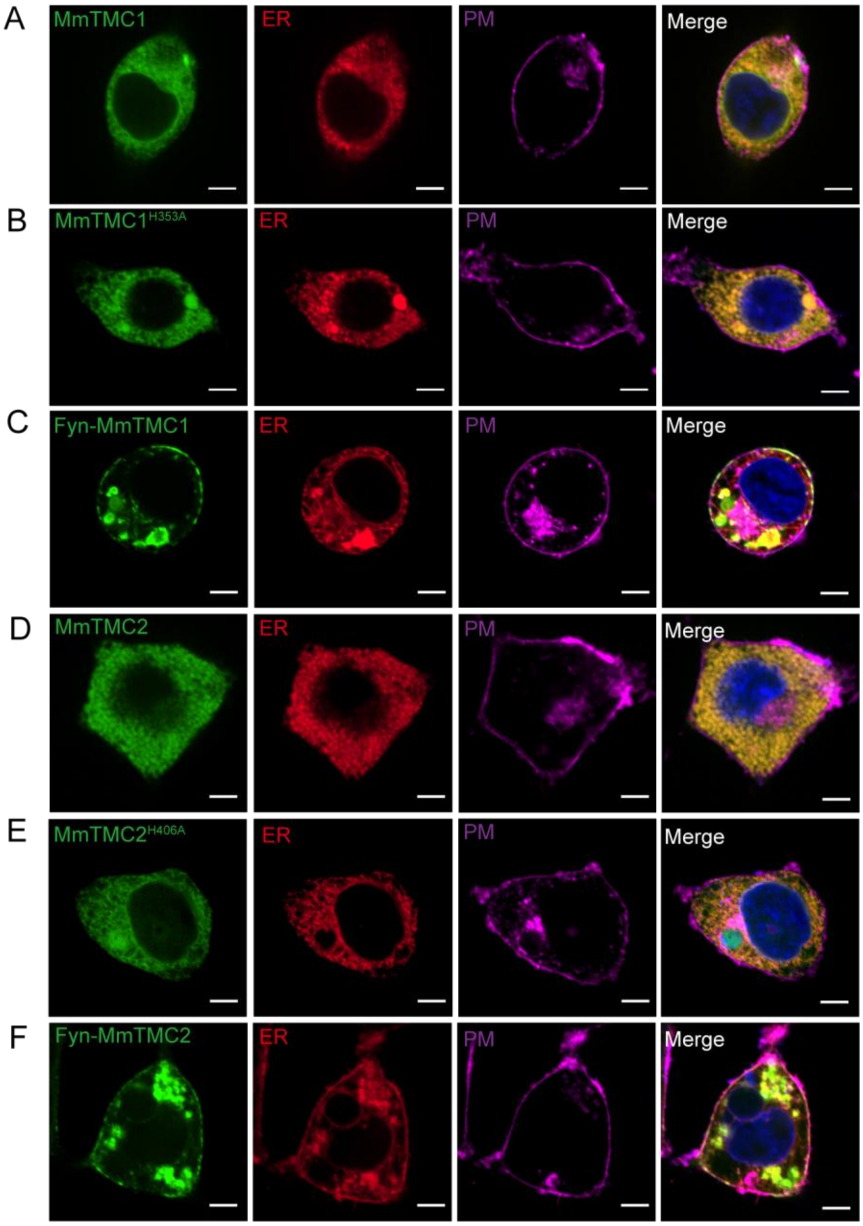

Figure S1. MmTMC1 and MmTMC2 do not localize to the plasma membrane in HEK 293T cells. (A–C): Representative fluorescent images of HEK 293T cells co-expressing WT MmTMC1-EGFP (A), MmTMC1^{H353A}-EGFP (B), and Fyn-MmTMC1-EGFP (C), along with an endoplasmic reticulum (ER) marker (red) and stained with CellMask™ plasma membrane (PM) stains (magenta). (D–F) Representative fluorescent images of HEK 293T cells co-expressing WT MmTMC2-EGFP (D), MmTMC2^{H406A}-EGFP (E), and Fyn-MmTMC2-EGFP (F), along with an ER marker (red) and stained with CellMask™ plasma membrane stains (magenta). The rightmost panels show merged images including Hoechst nuclear staining (blue). Scale bars, 5 µm.

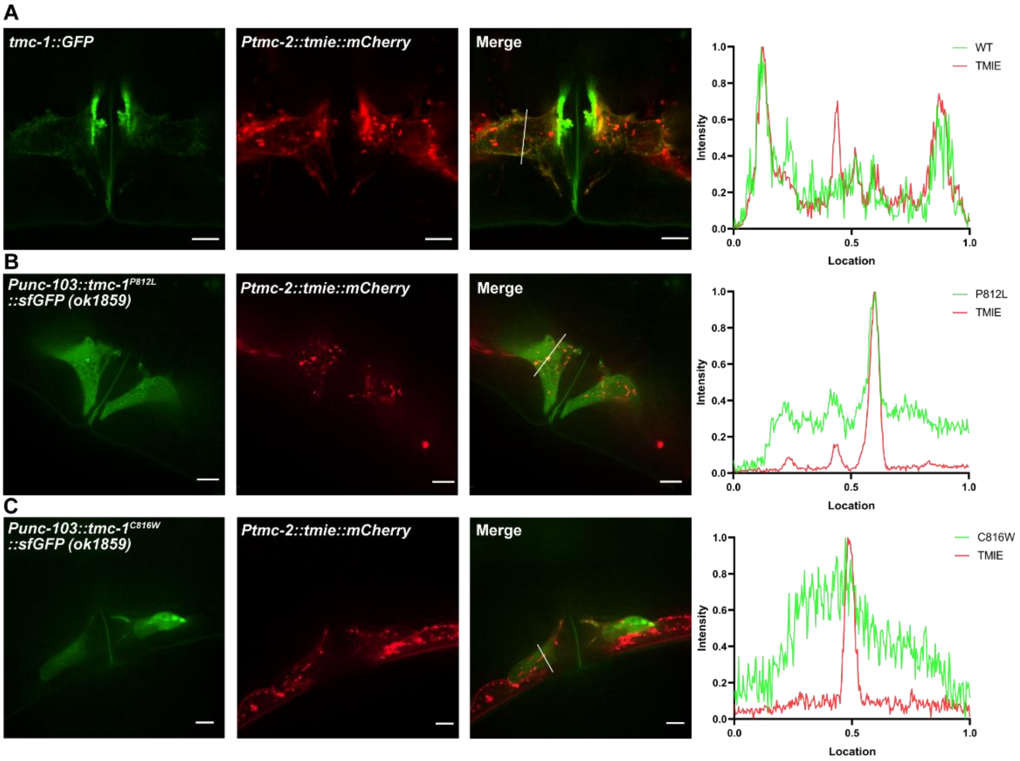

Figure S2 Mis-localized TMC-1 mutants interfere with the plasma membrane localization of its binding partner ceTMIE. (A-C) Representative fluorescence images of VM cells co-expressing red fluorescently labeled ceTMIE with WT ceTMC-1 (A), ceTMC-1^{P812L} (B), and ceTMC-1^{C816W} (C) mutants. Fluorescence intensity histograms for the corresponding images are shown on the right panels. Scale bars, 5 μm.

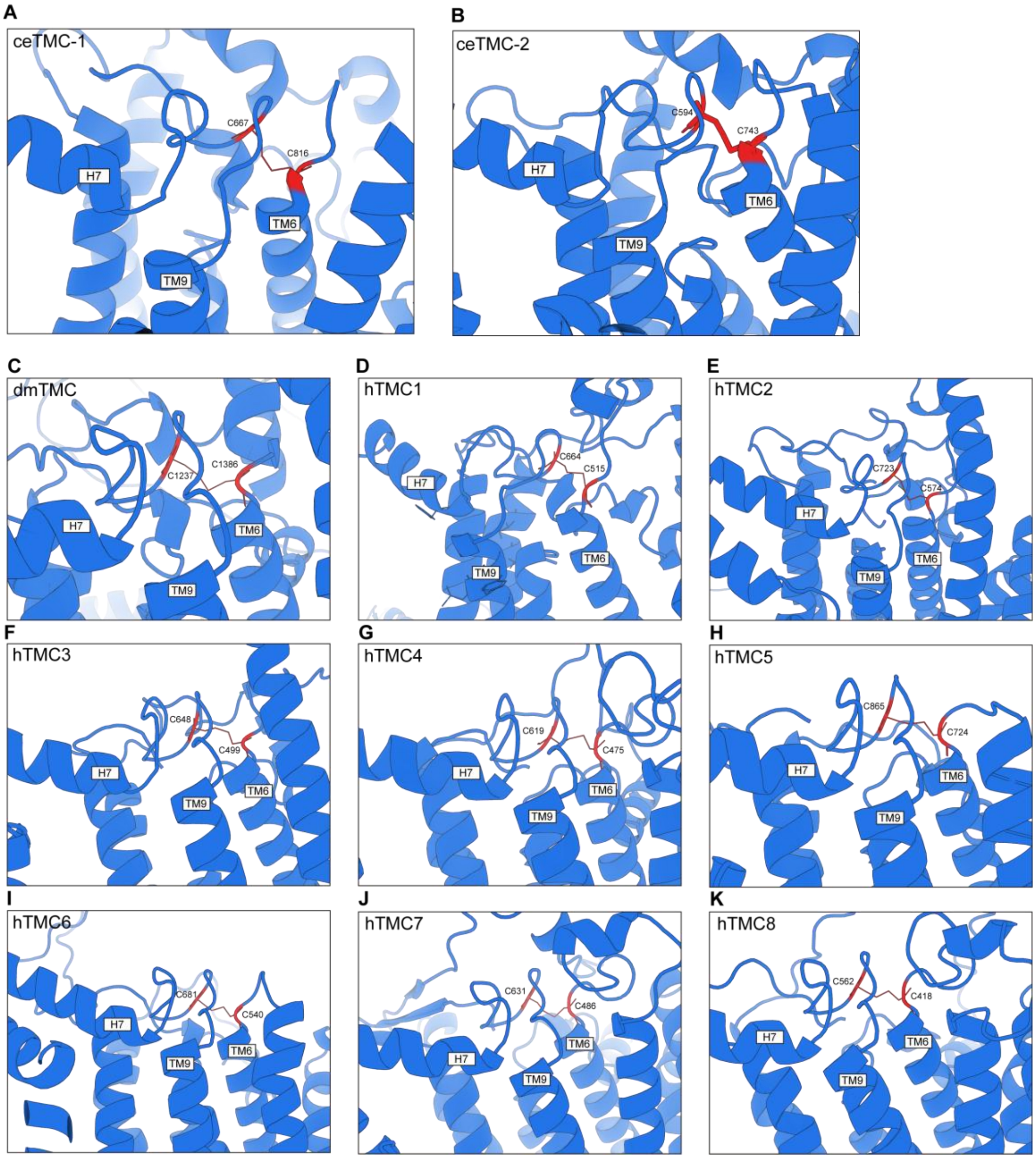

Figure S3 AlphaFold predictions of evolutionarily conserved disulfide bonds in TMC family members. (**A-B**) Cryo-EM structures of native *C. elegans* TMC-1 (**A**) and TMC-2 (**B**) reveal that two conserved functional hotspots are linked by disulfide bonds (highlighted in red). (**C-K**) AlphaFold-predicted tertiary structures of *Drosophila* TMC (**C**) and human TMC1-8 (**D-K**), with disulfide bonds highlighted in red.

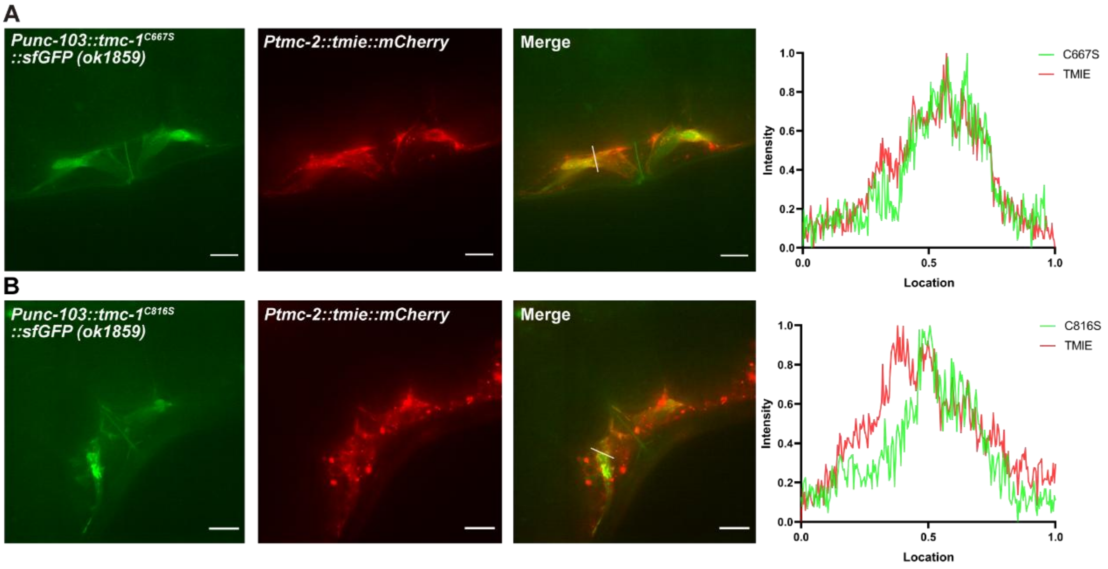

Figure S4 Disulfide-bond-disrupted TMC-1 mutants interfere with the plasma membrane localization of its binding partner ceTMIE. (A-B) Representative fluorescence images of VM cells co-expressing red fluorescently labeled ceTMIE with ceTMC-1^{C667S} (A) and ceTMC-1^{C816S} (B) mutants. Fluorescence intensity histograms for the corresponding images are shown on the right panels. Scale bars, 5 μm.

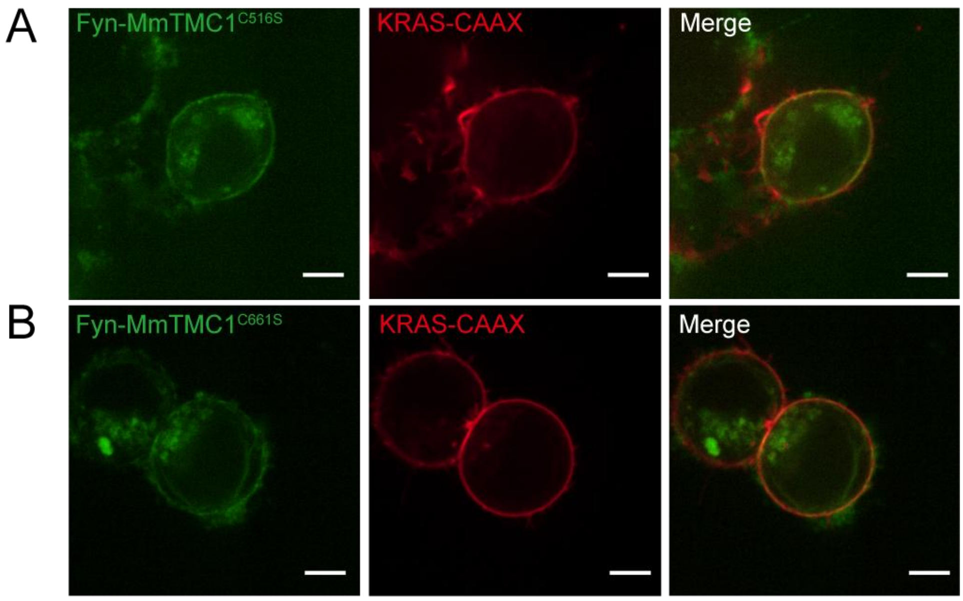

Figure S5 Fyn-tagged, disulfide-bond-disrupted MmTMC1 mutants localize to the plasma membrane in HEK 293T cells. (A-B) Representative fluorescent images of HEK 293T cells co-expressing Fyn-MmTMC1^{C516S}-EGFP (A) or Fyn-MmTMC1^{C661S}-EGFP (B) with the plasma membrane marker KRAS-CAAX (red). Scale bars, 5 μm.
